# Seasonality of birth defects in West Africa: could congenital Zika syndrome be to blame?

**DOI:** 10.12688/f1000research.13858.2

**Published:** 2018-07-19

**Authors:** Maimuna S. Majumder, Rosanna Hess, Ratchneewan Ross, Helen Piontkivska

**Affiliations:** 1 Computational Epidemiology Group, Division of Emergency Medicine, Boston Children's Hospital, Boston, MA, 02115, USA; 2 Engineering Systems Division, Massachusetts Institute of Technology, Cambridge, MA, 02142, USA; 3 Research for Health, Inc., Cuyahoga Falls, OH, 44223, USA; 4 School of Nursing, University of North Carolina at Greensboro, Greensboro, NC, 27408, USA; 5 241 Cunningham Hall, Department of Biological Sciences & School of Biomedical Sciences, Kent State University, Kent, OH, 44242, USA

**Keywords:** Zika virus; ZIKV; birth defects; microcephaly; congenital Zika syndrome; West Africa; seasonality

## Abstract

The link between Zika virus infection during pregnancy and microcephaly and other neurodevelopmental defects in infants, referred to as congenital Zika syndrome (CZS), was recently discovered. One key question that remains is whether such neurodevelopmental abnormalities are limited to the recently evolved Asiatic ZIKV strains or if they can also be induced by endemic African strains. Thus, we examined birth registries from one particular hospital from a country in West Africa, where ZIKV is endemic. Results showed a seasonal pattern of birth defects that is consistent with potential CZS, which corresponds to a range of presumed maternal infection that encompasses both the peak of the warm, rainy season as well as the months immediately following it, when mosquito activity is likely high. While we refrain from definitively linking ZIKV infection and birth defects in West Africa at this time, in part due to scant data available from the region, we hope that this report will initiate broader surveillance efforts that may help shed light onto mechanisms underlying CZS.

## Introduction

Since 2015, when an initial link between Zika Virus (ZIKV) and microcephaly was discovered in Brazil ^1, 2^, the term congenital Zika syndrome (CZS) has been coined to reflect a broad range of Zika-linked neurodevelopmental damages ^3^ beyond microcephaly ^3– 5^, including ocular ^6, 7^ and auditory defects ^8, 9^. The overall risk of Zika-linked birth defects has been estimated at ~10% and 15% for infections during the 1st trimester ^10^, potentially impacting thousands of infants in the US and US territories alone. Concerns exist that ZIKV-related outcomes are underreported, particularly when ZIKV infections result in neurodevelopmental abnormalities without visible microcephaly (e.g., developmental delays and learning disabilities that would not be immediately noticeable ^11– 13^). Such outcomes are likely not recorded, particularly if the causative infection was asymptomatic ^14^. Further, a recent CDC report that examined birth defect records from 15 US jurisdictions showed a statistically significant increase in prevalence of birth defects potentially consistent with CZS in areas with documented local ZIKV transmission in the second half of 2016 ^15^. Notably, the majority of infants and fetuses with birth defects potentially related to ZIKV infection in this report lacked ZIKV infection testing, which may be in part attributed to lack of known maternal exposure or other such indicators ^16^. Nonetheless, these findings are alarming and underscore not only the need for continued monitoring and surveillance, but also the need to better understand the full extent – as well as mechanisms – of neurodevelopmental defects associated with CZS.

## Can we expect to find cases of congenital Zika syndrome in the African Continent?

Currently our understanding of the mechanisms that underlie CZS remains limited, including the possibility that ZIKV infection is a necessary but insufficient condition for CZS ^17^. One key question is whether ZIKV-mediated birth defects are associated with a specific strain of ZIKV, which, for example, could have evolved after ZIKV migrated from South East Asia to French Polynesia and Brazil ^18^ and now spread to North America, including the US ^10, 15^. However, other studies suggest that African strains are likely to be as pathogenic as the Asiatic strains (e.g., 19, 20). Thus, the lack of connection between ZIKV infection in pregnancy and birth defects prior to the 2015–16 Brazilian outbreak may instead be attributable to the benign nature of ZIKV infection in adults ^21^ and lack of surveillance, including that infectious agents as well as surveillance of birth defects, among other factors. This can be illustrated by a report of a handful of birth defects in Hawaii in 2009–2012, which now has been shown to be associated with ZIVK infection, but was undetected as such until the Brazilian microcephaly epidemic brought ZIKV into the spotlight ^22^.

Earlier studies from the African Continent – where ZIKV is endemic – have documented a relatively high prevalence of ZIKV antibodies in human populations, including in West Africa (e.g., Nigeria ^23, 24^, Sierra-Leone ^25^, Senegal ^26^, Burkina Faso, Cote D'Ivoire, Guinea-Bissau, Cameroon, Mali, Niger, Benin, Gabon ^27^, supporting the premise that ZIKV is present across multiple countries in West Africa region (reviewed in Kindhauser *et al.* ^28^)). Despite this, there exists negligible data regarding CZS across the African Continent; nevertheless, lack of evidence should not be taken as definitive proof of absence ^19^. Thus, here we examine birth registries from one particular hospital in West Africa from a country considered by the WHO to be at medium risk of a ZIKV outbreak ^29^. The study was approved by the Committee on Administration of the hospital where the data was collected in lieu of a functioning Ethics Committee. But as there are ongoing security concerns in this location, that committee required that the hospital name and location be kept anonymous to ensure the safety of the hospital and staff. The chosen hospital has a full service maternity/OB department, staffed by well-trained qualified obstetricians, midwives, and obstetric nurses. It handled about 2000 births per year during the studied period; reports compiled from the hospital data are routinely used by the country’s government.

## Case study: seasonality of CZS-type birth defects in a hospital in West Africa

Risk of major neurodevelopmental defects, such as microcephaly, appears to be particularly high if vertical transmission occurs during the first trimester, especially within a “vulnerability window” around 12 weeks (10 to 14 weeks) post-conception ^10, 30^. Thus, we hypothesized that – similar to seasonal malaria infections, which peak a few weeks following abundant rainfalls during the “rainy” season, typically from August through October in the study region ^31, 32^ – seasonal variations in the number of CZS-type birth defects would be detectable from the aforementioned hospital data. Such expectations are consistent with prior findings from Senegal (West Africa) ^33^ and Kenya (East Africa) ^34^, where Rift Valley Fever epizootics were associated with heavy rainfalls. Our hypothesis was further informed by the temporal relationships between the number of ZIKV infections and microcephaly cases reported in Brazil ^35^. We expected that the peak of CZS-type birth defects (such as documented cases of microcephaly and/or stillbirth) would coincide with vertical ZIKV transmission at around 12-weeks post-conception during the peak of the rainy season, assuming an approximate 3-week lag between maternal infection and vertical transmission ^36^, as suggested by data from Brazil in 2015 ^37^.

## Methods

A total of 13445 birth registries (2009–2015) from a non-governmental hospital in West Africa were examined to determine whether we could identify a seasonal pattern of birth defects potentially attributable to ZIKV infection (i.e., CZS-type birth defects). The number of births and respective outcomes (i.e., live birth versus stillbirth) and reported complications (i.e., fetal malformation, breech, etc.) were collated by month/year. Reporting standards for birth complications varied between years; thus, we focused exclusively on visible neurodevelopmental complications (such as microcephaly) and pregnancy losses (such as stillbirth) that could be attributed to potential CZS ^3, 4^ ( Supplementary Table 1 and Supplementary Table 2). To infer the “vulnerability window” of 12 weeks (spanning 10 to 14 weeks) post-conception, we assumed that births that were not reported as premature in the records were full-term, thus enabling us to infer the likely month of conception ^35^.

We also considered national average monthly temperature and rainfall data for the study years, collected from the World Bank Climate Change Knowledge Portal database. These values were treated as proxy indicators for mosquito activity in the hospital catchment area at time of maternal infection. To visualize any relevant trends, we plotted these data, as well as the average percentage of birth defects consistent with potential CZS by month.

## Results and discussion

**Figure 1.**
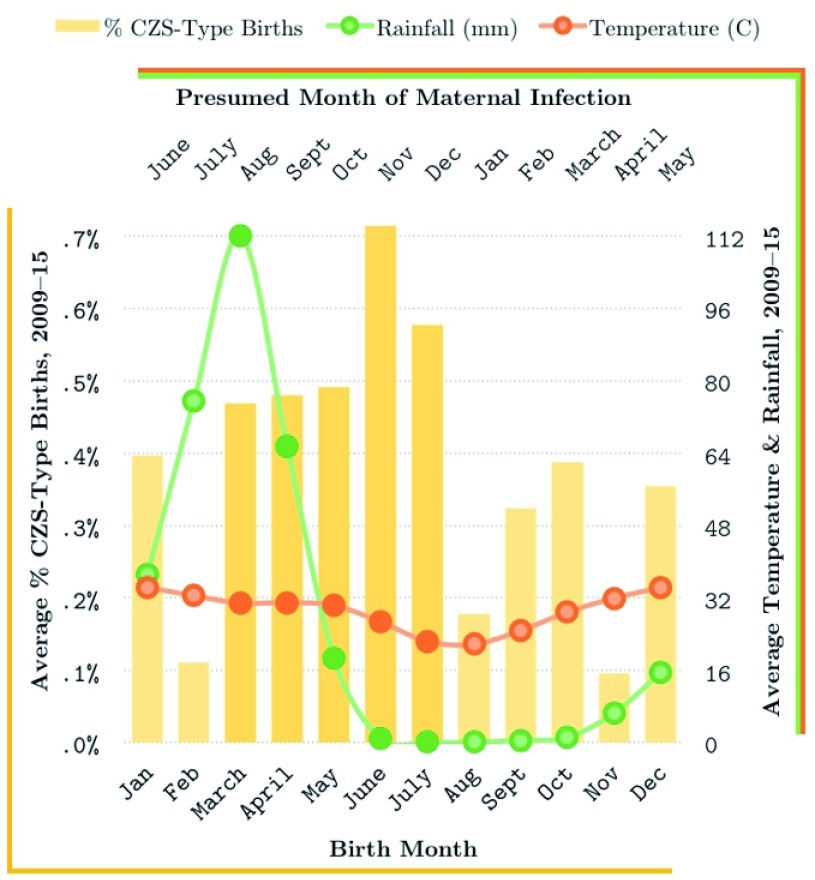
Average birth defects consistent with congenital Zika syndrome per month, with corresponding average daily temperatures and rainfall during presumed maternal month of infection. Bottom axis corresponds with the birth month depicted with yellow bars (i.e., with the left scale that shows % CZS defects), while the top axis corresponds with the month of maternal infection (and thus, with the rainfall and temperature shown on the left, depicted in green and red, respectively).

As shown in Figure 1, the average percentage of births consistent with potential CZS demonstrates a marked peak between March and July, which places maternal month of infection between August and December of the previous year. These months encompass the peak and latter half of the warm, rainy season (August–October) as well as the first half of the cool, dry season that immediately follows (October–December) in the study region, which likely represent months with considerable mosquito activity. Notably, the hospital from which these birth defects data were acquired also generally experiences a peak in childhood malaria cases every October, which falls squarely in the middle of the August to December range of presumed maternal month of ZIKV infection determined here.

With this in mind, the early months of the cool, dry season (October–December) are likely hospitable enough for mosquito vectors to thrive and spread pathogens, including ZIKV; this may explain why the range of our presumed maternal month of infection (August–December) extends past the rainy season, given that mosquitoes require a balanced environment for survival, including both moderate rainfall and optimal temperatures ^38^.

Our results suggest that a seasonal pattern exists with respect to CZS-type birth defects reported in the study region ( Figure 1), where the largest fraction of said defects appear to occur in the months of March through July. Furthermore, this pattern can be linked to ecological evidence, such as rainfall and temperature trends that likely facilitate maternal ZIKV infection. While consistent with the expectation that some of these defects might be attributable to (unreported) ZIKV infections that occurred early in pregnancy (and indeed resemble temporal patterns from studies in Brazil ^35^), our findings stop short of definitively linking ZIKV infection and birth defects in the study region, in part due to scant data. Instead, by reviewing the potential limitations of the data analyzed here, we hope that this report will initiate broader surveillance efforts – of both the infectious agents, including ZIKV, as well as that of birth defects - that may help shed light onto mechanisms that underlie CZS, including utilization of data that might already be available across various African countries where ZIKV is endemic and/or competent vectors exist (e.g., Gabon ^39^, Central African Republic ^40^). For example, a recent WHO Bulletin on Outbreaks and Other Emergencies ^41^ reported a number of microcephaly cases from Angola (a country listed in the High risk category ^29^) that appear to be linked to ZIKV infection, despite the lack of direct PCR confirmation from the specimens. Due to only recent implementation of active surveillance in the country, the true magnitude of the event is not yet clearly understood. Nonetheless, it is important for insights into a broader pattern of potential CZS defects, despite the lack of experimental ZIKV infection confirmation or lack of evidence of ongoing active ZIKV transmission in Angola (i.e. only two ZIKV cases were reported from Angola in early 2017 ^42^).

Several conservative assumptions made in this analysis, such as assuming a gestation period of ~9 months, or not classifying “low birth weights” (which may represent full-term births of ZIKV-infected fetuses) as a CZS-type birth defect, would likely lead to underestimation of potential trends, if any. We also assumed that the available birth records were representative of the pregnancy/birth patterns that occur across the entire region. Other limitations of the available data are related to the standard of care that is feasible in much of West Africa, including (i) lack of family history and/or genetic testing for mutations in loci responsible for primary microcephaly; (ii) lack of laboratory evidence or testing for ZIKV and/or other infections, including TORCH agents ^43, 44^, often due to inability to pay for testing (e.g., 45); and (iii) lack of detailed clinical prenatal history, including whether rash and/or other symptoms of Zika infection were present at any point during pregnancy. This final limitation may be considered minor, given that the majority of ZIKV infections are asymptomatic ^21, 30^. Additionally, no data were available regarding other clinically relevant factors that are also associated with microcephaly ^46^, such as history of excessive alcohol consumption or recreational drug use, and/or prolonged exposure to pesticides, such as pyriproxyfen. However, the former life-style factors are unlikely to have a seasonal effect spanning several years, and the role of the latter factor as a causative agent of microcephaly remains unclear ^47^. There is also a lack of precise ecological data, including estimates of rainfalls in the hospital catchment area, the distribution and feeding habits of mosquitoes, and whether or not said mosquitoes carry ZIKV, as well as data regarding ZIKV prevalence in the human population.

Despite these limitations, our findings suggest that using the data we already have – even in the absence of formal surveillance systems for CZS – can provide compelling, introductory insights. In the future, work that employs existing data from hospitals across the African continent – which encompasses countries with a variety of climates, dry and rainy seasons, and suitability for widespread mosquito habitats – should be pursued.

## Data availability

The data referenced by this article are under copyright with the following copyright statement: Copyright: © 2018 Majumder MS et al.

Data associated with the article are available under the terms of the Creative Commons Zero "No rights reserved" data waiver (CC0 1.0 Public domain dedication). 

Figshare : Data for Figure 1. Average birth defects consistent with congenital Zika syndrome per month, with corresponding average daily temperatures and rainfall during presumed maternal month of infection. doi: 10.6084/m9.figshare.5387029. Data are available under the terms of the Creative Commons Attribution 4.0 International license (CC-BY 4.0).

## Supplementary material

Supplemental Table 1. A list of birth complications (as recorded in the health records) consistent with potential congenital Zika syndrome.

Supplemental Table 2. A list of birth complications (as recorded in the health records) that are not consistent with potential congenital Zika syndrome and/or missing.
