# Supplementary material for: Seasonality of birth defects in West Africa: could congenital Zika syndrome be to blame?
Source: F1000Res. 2018 Jul 19;7:159. Originally published 2018 Feb 7. [Version 2] doi: 10.12688/f1000research.13858.2 (PMC6058464; doi:10.12688/f1000research.13858.2)
Supplement: Peer review history

# Peer review: Referee response for version 2

**DOI:** 10.5256/f1000research.17079.r36769

**Published:** 2018-08-03

**Authors:** Matthew T. Aliota

**Affiliations:** 1 University of Minnesota, Minneapolis, MN, USA

The authors have addressed my concerns adequately.

I have read this submission. I believe that I have an appropriate level of expertise to confirm that it is of an acceptable scientific standard.

# Peer review: Referee response for version 2

**DOI:** 10.5256/f1000research.17079.r36262

**Published:** 2018-07-23

**Authors:** Diana Valencia

**Affiliations:** 1 Centers for Disease Control and Prevention (CDC), Atlanta, GA, USA

The  authors addressed all my concerns, and have no further comments.

I have read this submission. I believe that I have an appropriate level of expertise to confirm that it is of an acceptable scientific standard.

# Peer review: Referee response for version 1

**DOI:** 10.5256/f1000research.15063.r35015

**Published:** 2018-06-25

**Authors:** Catherine M. Brown

**Affiliations:** 1 Massachusetts Department of Public Health (MDPH), Boston, MA, USA

The authors present an analysis of birth defects by season from a single hospital in an unnamed West African country relative to a purported mosquito season. Their conclusion is that the seasonality of certain types of birth defects, consistent with CZS, could be explain by Zika virus transmission in the area. This is an interesting attempt to use available data to speculate about the impact of Zika virus on congenital defects in an endemic area. Although this is certainly thought-provoking and supports the authors recommendation to improve surveillance for Zika virus in endemic areas, there is insufficient discussion of all of the limitations to this particular analysis which, if included, would cast doubt on their proposed conclusion.

 The limitation that the data are from a single hospital in an un-named location make it impossible to make any generalizable conclusions  and to understand any limitations completely.

 Data do not exist on the underlying sero-prevalence of Zika virus infection in this population (I assume). However, given that Zika virus infection likely results in lifelong immunity, one might not expect to see any change in CZS-like defects simply due to a low number of susceptible pregnant women. Were the women with infants with birth defects generally younger and therefore less likely to have been previously infected? And why wouldn't we expect children to have a high rate of infection resulting in immunity as they reach child-bearing age?

 Again, although difficult to determine given the lack of geographic specificity provided, there is minimal discussion in the paper of other potential causes of microcephaly; most notably rubella and malnutrition. Although rubella-related birth defects might not be expected to show any seasonality, malnutrition might. As might toxoplasmosis.

 The time period covered by this report encompasses both the Ebola outbreak and significant Yellow fever activity; both of these could cause confounding in the data.

 Mosquito species are quite variable in their feeding habits, optimal climactic conditions and in their vectorial capacity. Relying on patterns of malarial disease to represent what is likely to happen with a flavivirus like Zika which is transmitted by different species of mosquitoes should be considered to be a limitation. What about using Yellow fever or dengue disease patterns?

 Lastly, while I understand the argument the authors are making, I found the assertion that the maternal infection period of August through October encompassed the peak and latter half of the warm, rainy season confusing. Based on the presented data, August through October had minimal rain and lower than average temperatures. October through December was described as the cool, dry season although temperatures peak for the year in November through January (according to the data presented by the authors).

I have read this submission. I believe that I have an appropriate level of expertise to confirm that it is of an acceptable scientific standard, however I have significant reservations, as outlined above.

# Response

**Published:** 2018-07-16

**Authors:** Helen Piontkivska

**Affiliations:** Kent State University

*>>>The limitation that the data are from a single hospital in an un-named location make it impossible to make any generalizable conclusions and to understand any limitations completely.*

We appreciate these limitations, and they were requirements for us to access the data. Specifically, the Committee on Administration of the hospital required us not to name the hospital or the country. We added a statement clarifying this. Our goal was to spur further discussion and further research from larger sample sizes in multiple locations in order to access the generality of this pattern.

*>>> Data do not exist on the underlying sero-prevalence of Zika virus infection in this population (I assume). However, given that Zika virus infection likely results in lifelong immunity, one might not expect to see any change in CZS-like defects simply due to a low number of susceptible pregnant women. Were the women with infants with birth defects generally younger and therefore less likely to have been previously infected? And why wouldn't we expect children to have a high rate of infection resulting in immunity as they reach child-bearing age?*

It is still unclear whether in humans (versus non-human primates) Zika infection confers life-long immunity (Siedner, Ryan and Bogoch 2018). However, age distribution of mothers of infants with CZS-like birth defects indicates that such birth defects appear across multiple age cohorts, from 15 to 37, with the mean number of CZS-like cases per each age group of 2.39 (± standard error of 0.42). Further research of larger cohorts across different locations is needed to definitively test whether there are any maternal-age related effects.

*>>> Again, although difficult to determine given the lack of geographic specificity provided, there is minimal discussion in the paper of other potential causes of microcephaly; most notably rubella and malnutrition. Although rubella-related birth defects might not be expected to show any seasonality, malnutrition might. As might toxoplasmosis. The time period covered by this report encompasses both the Ebola outbreak and significant Yellow fever activity; both of these could cause confounding in the data.*

We agree that there are many human health issues that could display seasonality. This underscores the need for widespread comprehensive test for infectious agents and prenatal care, although implementation of such tests in Africa will require significant investments to be feasible.

*>>> Mosquito species are quite variable in their feeding habits, optimal climactic conditions and in their vectorial capacity. Relying on patterns of malarial disease to represent what is likely to happen with a flavivirus like Zika which is transmitted by different species of mosquitoes should be considered to be a limitation. What about using Yellow fever or dengue disease patterns?*

In our inferences we did not rely on the malaria pattern to infer seasonality; instead, it was referenced to indicate consistency in the inferred activities from the temperature and rainfall data. Regretfully, no data regarding yellow fever or dengue infections were available. However, the temperature ranges of the peak transmission of malaria and dengue are overlapping [mid 20's ºC for malaria, per (Beck-Johnson et al. 2013); 23-34ºC for DENV, per (Mordecai et al. 2017)]. Therefore, we expect that the transmission patterns would be similar.

Beck-Johnson, L. M., et al. (2013). "The effect of temperature on Anopheles mosquito population dynamics and the potential for malaria transmission." PLoS One **8**(11): e79276.

Mordecai, E. A., et al. (2017). "Detecting the impact of temperature on transmission of Zika, dengue, and chikungunya using mechanistic models." PLoS Negl Trop Dis **11**(4): e0005568.

*>>> Lastly, while I understand the argument the authors are making, I found the assertion that the maternal infection period of August through October encompassed the peak and latter half of the warm, rainy season confusing. Based on the presented data, August through October had minimal rain and lower than average temperatures. October through December was described as the cool, dry season although temperatures peak for the year in November through January (according to the data presented by the authors).*

We revised the figure 1 legend to make it clear that the bottom axis corresponds with the birth month (depicted with yellow bars) and therefore with the left Y axis that shows % CZS-type birth defects, while the top axis corresponds with the month of maternal infection (i.e., with the rainfall and temperature shown on the left, depicted in green and red, respectively).  We have also added color guides to the figure to make it visually clear.

# Peer review: Referee response for version 1

**DOI:** 10.5256/f1000research.15063.r35010

**Published:** 2018-06-25

**Authors:** Diana Valencia

**Affiliations:** 1 Centers for Disease Control and Prevention (CDC), Atlanta, GA, USA

Thank you for the opportunity to review this article. I have the following questions/suggestions for the consideration of the authors:

1. The following important reference is missing from the article, please include: Rasmussen et al. (2016) ^1^
2. Authors should mention that Zika migrated not only to Brazil but to the Americas.
3. What are the characteristics of the chosen hospital? Why only one hospital was chosen? Does the hospital has a good reliable birth defect registry?
4. There is no description of the birth defects taken into account for the paper; which CZS-type birth defects were chosen form the hospital registry? Were the birth defects confirmed? Was any description available in medical records? Were X-rays available for review? 
5. I agree with the authors on the importance to have birth defects surveillance systems in place. I hope that this article will contribute to the awareness of public health authorities regarding this important issue. 
6. Birth defects surveillance is needed to identify Zika related defects. As the authors mention, the majority of infected pregnant women are asymptomatic.
7. I think it will be important to differentiate in the article between Zika virus surveillance and birth defects surveillance, and the difficulties of implementing them in Africa.

I have read this submission. I believe that I have an appropriate level of expertise to confirm that it is of an acceptable scientific standard, however I have significant reservations, as outlined above.

## References

[1] RasmussenSAJamiesonDJHoneinMAPetersenLR: Zika Virus and Birth Defects--Reviewing the Evidence for Causality. *N Engl J Med*.2016;374(20) : 10.1056/NEJMsr1604338 1981-7 10.1056/NEJMsr1604338 27074377

# Response

**Published:** 2018-07-16

**Authors:** Helen Piontkivska

**Affiliations:** Kent State University

*>>> 1.  The following important reference is missing from the article, please include: Rasmussen et al. (2016)*

Thank you for pointing out our oversight. We added this important reference to the paper.

*>>>> 2. Authors should mention that Zika migrated not only to Brazil but to the Americas.*

Done.  

*>>>> 3. What are the characteristics of the chosen hospital? Why only one hospital was chosen? Does the hospital has a good reliable birth defect registry?*

Additional identifiable characteristics were not included in order to protect the identity of the hospital; however, we now added several broad characteristics of the hospital to the manuscript.

These were the only data that were available to us, and we are confident in the quality of the birth defect registry. We are excited that we were able to demonstrate this trend even with the relatively small sample available to us. We hope this study will spur further comprehensive analyses of larger datasets.

*>>> 4.  There is no description of the birth defects taken into account for the paper; which CZS-type birth defects were chosen form the hospital registry? Were the birth defects confirmed? Was any description available in medical records? Were X-rays available for review?*

The description of birth complications that we categorize as a CZS-type (i.e., birth defects consistent with potential congenital Zika syndrome) are listed in the Supplementary table 1, while Supplementary table 2 lists the non-CZS-type complications. We did not have access to individuals' medical records beyond the descriptions listed in Supplementary tables. The hospital does not routinely perform X-rays on newborns with birth defects; thus, no further description of cases or X-rays were available to us.

*>>> 5. I agree with the authors on the importance to have birth defects surveillance systems in place. I hope that this article will contribute to the awareness of public health authorities regarding this important issue.*

*6. Birth defects surveillance is needed to identify Zika related defects. As the authors mention, the majority of infected pregnant women are asymptomatic.*

Thank you, we completely agree.

*>>> 7.  I think it will be important to differentiate in the article between Zika virus surveillance and birth defects surveillance, and the difficulties of implementing them in Africa.*

We agree, and have added statements to that effect. Enhanced birth defect surveillance within hospitals are potentially feasible with the current resources, although they may require additional personnel and/or training. However, the surveillance of birth defects outside of hospital settings would be problematic, though potentially possible. On the other hand, Zika virus surveillance per se, as well as surveillance of other viral diseases, would require substantial financial investments globally, as well as overcoming other challenges related to the implementation of such surveillance.

# Peer review: Referee response for version 1

**DOI:** 10.5256/f1000research.15063.r32204

**Published:** 2018-04-03

**Authors:** Matthew T. Aliota

**Affiliations:** 1 University of Minnesota, Minneapolis, MN, USA

The authors have produced a paper speculating that African lineage Zika virus has the same capacity to cause congenital Zika syndrome (CZS) as more recent Asian/American outbreak isolates. This was accomplished by analyzing birth outcome reports from one hospital in West Africa and temporally linking outcomes consistent with CZS to seasonal patterns when mosquito populations would be abundant. The paper is well written and certainly addresses an outstanding question in the field. In sum, this article's major purpose is to create a discussion and provide a rationale for broader Zika surveillance activities in Africa, which I I believe has been accomplished, however, there are a few minor details that could be changed to facilitate understanding by the reader.

1. I understand the need for security and thus not naming the hospital but would naming the country truly jeopardize security?
2. While I agree that it is possible that African ZIKV has always had the capability of causing CZS and that it is likely underreported or unrecognized, an alternative explanation is that in Africa where ZIKV is endemic girls and women are exposed early in life and subsequent immunity provides protection against CZS during child bearing years. Is there any age distribution that can be associated with the outcomes presented here?
3. Throughout comparisons are made to Malaria which is transmitted by Anopheles mosquitoes, whereas, ZIKV is likely transmitted by an Aedes species mosquito. Therefore, the same environmental factors may not drive transmission of both equally. Do the authors have any data on, for example, dengue cases that were reported at the same hospital during the study period? 
4. The data are from a country that is at "medium risk for a Zika outbreak". I believe it is more important to know what the estimated seroprevalence of Zika exposure is in this country. This suggests that perhaps Zika is not endemic in the country.

I have read this submission. I believe that I have an appropriate level of expertise to confirm that it is of an acceptable scientific standard, however I have significant reservations, as outlined above.

# Response

**Published:** 2018-07-16

**Authors:** Helen Piontkivska

**Affiliations:** Kent State University

*>>> 1. I understand the need for security and thus not naming the hospital but would naming the country truly jeopardize security?*

We agree with you in principle; however, the Committee on Administration of the hospital required us not to name the hospital or the country. We added a statement clarifying this.  

*>>> 2. While I agree that it is possible that African ZIKV has always had the capability of causing CZS and that it is likely underreported or unrecognized, an alternative explanation is that in Africa where ZIKV is endemic girls and women are exposed early in life and subsequent immunity provides protection against CZS during child bearing years. Is there any age distribution that can be associated with the outcomes presented here?*

We agree with the reviewer that the question of whether early exposure to ZIKV and/or other flaviviruses may offer subsequent long-term protection against CZS during childbearing years warrants further attention. CZS-type defects analyzed in our study were found in women of ages 15-37 years old; the average number of cases per maternal age class is 2.39 (± standard error of 0.42). Thus, these data are not well suited to test this hypothesis, which we hope can be tested in the future with appropriate power.  

*>>> 3. Throughout comparisons are made to Malaria which is transmitted by Anopheles mosquitoes, whereas, ZIKV is likely transmitted by an Aedes species mosquito. Therefore, the same environmental factors may not drive transmission of both equally. Do the authors have any data on, for example, dengue cases that were reported at the same hospital during the study period?*

There are no data on dengue cases available from the hospital. However, the temperature ranges of the peak transmission of malaria and dengue are overlapping [mid 20's ºC for malaria, per (Beck-Johnson et al. 2013); 23-34ºC for DENV, per (Mordecai et al. 2017)]. Therefore, we expect that the transmission patterns would be similar.

Beck-Johnson, L. M., et al. (2013). "The effect of temperature on Anopheles mosquito population dynamics and the potential for malaria transmission." PLoS One 8(11): e79276.

Mordecai, E. A., et al. (2017). "Detecting the impact of temperature on transmission of Zika, dengue, and chikungunya using mechanistic models." PLoS Negl Trop Dis 11(4): e0005568.

*>>> 4. The data are from a country that is at "medium risk for a Zika outbreak". I believe it is more important to know what the estimated seroprevalence of Zika exposure is in this country. This suggests that perhaps Zika is not endemic in the country.*

Unfortunately, there are relatively scant data available from the region, most of which comes from the decades-old studies. Specifically, antibodies to ZIKV were found in human blood samples collected in 1960-ies in Senegal, Burkina Faso, Cote D'Ivoire, Guinea-Bissau, Cameroon, Mali, Niger, Benin, Gabon (Brès 1970), in Senegal (Renaudet et al. 1978) and Nigeria (Adekolu-John and Fagbami 1983) in 1970-ies and 1980-ies, supporting the premise that ZIKV is present across multiple countries in West Africa region (reviewed in  (Kindhauser et al. 2016)). We hope that this study will motivate the collection of such data in the future. We added these references to the manuscript, to the "Can we expect to find cases of congenital Zika syndrome in the African Continent?" section.

Adekolu-John, E. O. and A. H. Fagbami (1983). "Arthropod-borne virus antibodies in sera of residents of Kainji Lake Basin, Nigeria 1980." Trans R Soc Trop Med Hyg **77**(2): 149-151.

Brès, P. (1970). "Données récentes apportées par les enquêtes sérologiques sur la prévalence des arbovirus en Afrique, avec référence spéciale à la fièvre jaune [Recent data from serological surveys on the prevalence of arbovirus infections in Africa, with special reference to yellow fever]." Bulletin of the World Health Organization **43**(2): 223-267.

Kindhauser, M. K., et al. (2016). "Zika: the origin and spread of a mosquito-borne virus." Bull World Health Organ **171082**.

Renaudet, J., et al. (1978). "[A serological survey of arboviruses in the human population of Senegal]." Bull Soc Pathol Exot Filiales **71**(2): 131-140.
